# *N*-(4-Chloro­phen­yl)benzamide

**DOI:** 10.1107/S1600536808008155

**Published:** 2008-03-29

**Authors:** B. Thimme Gowda, Miroslav Tokarčík, Jozef Kožíšek, B. P. Sowmya, Hartmut Fuess

**Affiliations:** a Department of Chemistry, Mangalore University, Mangalagangotri 574 199, Mangalore, India; b Faculty of Chemical and Food Technology, Slovak Technical University, Radlinského 9, SK-812 37 Bratislava, Slovak Republic; c Institute of Materials Science, Darmstadt University of Technology, Petersenstrasse 23, Darmstadt, D-64287, Germany

## Abstract

The structure of the title compound, C_13_H_10_ClNO, resembles those of *N*-phen­ylbenzamide, *N*-(2-chloro­phenyl)­benzamide and other benzanilides, with similar bond parameters. The amide group –NHCO– makes a dihedral angle of 29.95 (9)° with the benzoyl ring, while the benzoyl and aniline rings form a dihedral angle of 60.76 (3)°. The structure shows both intra- and inter­molecular hydrogen bonding. The mol­ecules are linked by N—H⋯O hydrogen bonds into chains running along the [100] direction.

## Related literature

For related literature, see: Gowda *et al.* (2003 ▶, 2007 ▶, 2008 ▶).
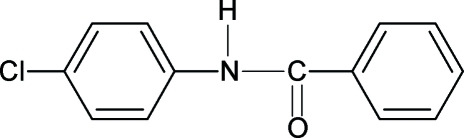


## Experimental

### 

#### Crystal data

C_13_H_10_ClNO
*M* *_r_* = 231.67
Triclinic, 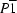

*a* = 5.3789 (1) Å
*b* = 7.8501 (2) Å
*c* = 13.6318 (4) Å
α = 106.509 (2)°
β = 98.380 (2)°
γ = 90.631 (2)°
*V* = 545.15 (2) Å^3^
*Z* = 2
Mo *K*α radiation
μ = 0.33 mm^−1^
*T* = 295 (2) K
0.52 × 0.25 × 0.08 mm

#### Data collection

Oxford Xcalibur diffractometer
Absorption correction: analytical [*CrysAlis RED* (Oxford Diffraction, 2007 ▶), using a multifaceted crystal model based on expressions derived by Clark & Reid (1995 ▶)] *T*_min_ = 0.852, *T*_max_ = 0.975
23656 measured reflections
2087 independent reflections
1773 reflections with *I* > 2σ(*I*)
*R*_int_ = 0.026

#### Refinement

*R*[*F* ^2^ > 2σ(*F* ^2^)] = 0.032
*wR*(*F* ^2^) = 0.091
*S* = 1.08
2087 reflections
148 parameters
1 restraint
H atoms treated by a mixture of independent and constrained refinement
Δρ_max_ = 0.19 e Å^−3^
Δρ_min_ = −0.23 e Å^−3^

### 

Data collection: *CrysAlis CCD* (Oxford Diffraction, 2007 ▶); cell refinement: *CrysAlis RED* (Oxford Diffraction, 2007 ▶); data reduction: *CrysAlis RED*; program(s) used to solve structure: *SHELXS97* (Sheldrick, 2008 ▶); program(s) used to refine structure: *SHELXL97* (Sheldrick, 2008 ▶); molecular graphics: *ORTEP-3* (Farrugia, 1997 ▶) and *DIAMOND* (Brandenburg, 2002 ▶); software used to prepare material for publication: *SHELXL97*, *PLATON* (Spek, 2003 ▶) and *WinGX* (Farrugia, 1999 ▶).

## Supplementary Material

Crystal structure: contains datablocks I, global. DOI: 10.1107/S1600536808008155/dn2327sup1.cif

Structure factors: contains datablocks I. DOI: 10.1107/S1600536808008155/dn2327Isup2.hkl

Additional supplementary materials:  crystallographic information; 3D view; checkCIF report

## Figures and Tables

**Table 1:** Hydrogen-bond geometry (Å, °)

*D*—H⋯*A*	*D*—H	H⋯*A*	*D*⋯*A*	*D*—H⋯*A*
C9—H9⋯O1	0.93	2.43	2.9090 (17)	112
N1—H1N⋯O1^i^	0.845 (16)	2.390 (16)	3.1710 (15)	154.0 (15)
C13—H13⋯O1^i^	0.93	2.58	3.2507 (11)	129

Symmetry code: (i) 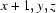.

**Fig. 1.**
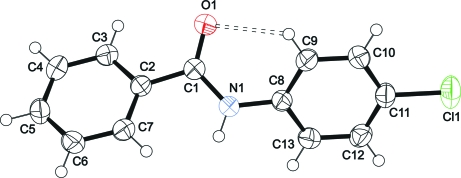
Molecular structure of the title compound showing the atom labelling scheme. Displacement ellipsoids are drawn at the 50% probability level. H atoms are represented as small spheres of arbitrary radii. Intramolecular hydrogen bond C9—H9···O1 is shown by dashed line.

**Fig. 2.**
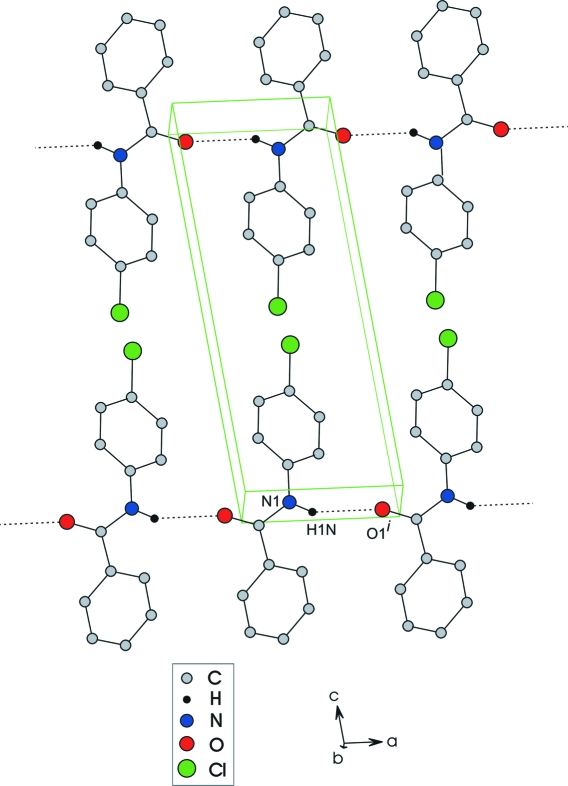
Part of the crystal structure of the title compound viewed down the b axis and showing infinite molecular chains in the[100] direction. H atoms not involved in intermolecular bonding have been omitted. [Symmetry code: (i) x + 1, y, z]

## supplementary crystallographic information

### Comment

In the present work, the structure of *N*-(4-chlorophenyl)benzamide (N4CPBA) has been determined to study the effect of substituents on the structures of benzanilides (Gowda *et al.*, 2003, 2007, 2008).

The structure of N4CPBA (Fig.1) is similar to those of *N*-(phenyl)benzamide, *N*-(2-chlorophenyl)benzamide, *N*-(3-chlorophenyl)benzamide and *N*-(4-methylphenyl)benzamide and other benzanilides (Gowda *et al.*, 2003, 2007, 2008). The amide group –NHCO– forms dihedral angle of 29.95 (9)° with the benzoyl ring, while the two benzene rings (benzoyl and aniline rings) form dihedral angle of 60.76 (3)°. Part of the structure of N4CPBA as viewed down the *b* axis and showing infinite molecular chains in the [100] direction is shown in Fig. 2. The chains are generated by the intermolecular N—H···O hydrogen bonds (Table 1).

### Experimental

The title compound was prepared according to the literature method (Gowda *et al.*, 2003). The purity of the compound was checked by determining its melting point. It was characterized by recording its infrared and NMR spectra. Single crystals of the title compound were obtained from an ethanolic solution and used for X-ray diffraction studies at room temperature.

### Refinement

H atoms attached to C atoms were placed in calculated positions and subsequently treated as riding with C—H distance 0.93 Å. H atom of the amide group was refined with the N—H distance restrained to 0.86 (4) Å. The *U*_iso_(H) values were set at 1.2 *U*_eq_(C,N).

### Crystal data

**Table:**

C_13_H_10_ClNO	*Z* = 2
*M**_r_* = 231.67	*F*_000_ = 240
Triclinic, *P*1	*D*_x_ = 1.411 Mg m^−^^3^
Hall symbol: -P 1	Mo *K*α radiation λ = 0.71073 Å
*a* = 5.3789 (1) Å	Cell parameters from 13860 reflections
*b* = 7.8501 (2) Å	θ = 3.1–29.3º
*c* = 13.6318 (4) Å	µ = 0.33 mm^−^^1^
α = 106.509 (2)º	*T* = 295 (2) K
β = 98.380 (2)º	Block, colorless
γ = 90.631 (2)º	0.52 × 0.25 × 0.08 mm
*V* = 545.15 (2) Å^3^	

### Data collection

**Table:**

Oxford Xcalibur diffractometer	2087 independent reflections
Radiation source: fine-focus sealed tube	1773 reflections with *I* > 2σ(*I*)
Monochromator: graphite	*R*_int_ = 0.026
Detector resolution: 10.434 pixels mm^-1^	θ_max_ = 25.8º
*T* = 295(2) K	θ_min_ = 5.6º
φ scans, and ω scans with κ offsets	*h* = −6→6
Absorption correction: analytical[CrysAlis RED (Oxford Diffraction, 2007). Analytical absorption correction using a multifaceted crystal model based on expressions derived by Clark & Reid (1995)]	*k* = −9→9
*T*_min_ = 0.852, *T*_max_ = 0.975	*l* = −16→16
23656 measured reflections	

### Refinement

**Table:**

Refinement on *F*^2^	Secondary atom site location: difference Fourier map
Least-squares matrix: full	Hydrogen site location: inferred from neighbouring sites
*R*[*F*^2^ > 2σ(*F*^2^)] = 0.032	H atoms treated by a mixture of independent and constrained refinement
*wR*(*F*^2^) = 0.091	*w* = 1/[σ^2^(*F*_o_^2^) + (0.0494*P*)^2^ + 0.0939*P*] where *P* = (*F*_o_^2^ + 2*F*_c_^2^)/3
*S* = 1.08	(Δ/σ)_max_ = 0.001
2087 reflections	Δρ_max_ = 0.19 e Å^−^^3^
148 parameters	Δρ_min_ = −0.23 e Å^−^^3^
1 restraint	Extinction correction: none
Primary atom site location: structure-invariant direct methods	

### Special details

**Table:**

Geometry. All e.s.d.'s (except the e.s.d. in the dihedral angle between two l.s. planes) are estimated using the full covariance matrix. The cell e.s.d.'s are taken into account individually in the estimation of e.s.d.'s in distances, angles and torsion angles; correlations between e.s.d.'s in cell parameters are only used when they are defined by crystal symmetry. An approximate (isotropic) treatment of cell e.s.d.'s is used for estimating e.s.d.'s involving l.s. planes.
Refinement. Refinement of *F*^2^ against ALL reflections. The weighted *R*-factor *wR* and goodness of fit *S* are based on *F*^2^, conventional *R*-factors *R* are based on *F*, with *F* set to zero for negative *F*^2^. The threshold expression of *F*^2^ > σ(*F*^2^) is used only for calculating *R*-factors(gt) *etc*. and is not relevant to the choice of reflections for refinement. *R*-factors based on *F*^2^ are statistically about twice as large as those based on *F*, and *R*- factors based on ALL data will be even larger.

### Fractional atomic coordinates and isotropic or equivalent isotropic displacement parameters (Å^2^)

**Table:**

	*x*	*y*	*z*	*U*_iso_*/*U*_eq_	
N1	0.3126 (2)	0.77177 (16)	0.02982 (9)	0.0381 (3)	
H1N	0.443 (3)	0.757 (2)	0.0010 (13)	0.046*	
O1	−0.11350 (19)	0.74813 (17)	0.00041 (8)	0.0563 (3)	
C1	0.0870 (2)	0.71549 (18)	−0.03078 (10)	0.0370 (3)	
C2	0.0976 (2)	0.61312 (17)	−0.14032 (10)	0.0343 (3)	
C3	−0.1018 (2)	0.62387 (19)	−0.21496 (11)	0.0396 (3)	
H3	−0.2346	0.6941	−0.1956	0.047*	
C4	−0.1045 (3)	0.5314 (2)	−0.31747 (11)	0.0450 (3)	
H4	−0.2376	0.5409	−0.3671	0.054*	
C5	0.0893 (3)	0.4249 (2)	−0.34662 (11)	0.0460 (4)	
H5	0.0871	0.3621	−0.4158	0.055*	
C6	0.2867 (3)	0.41171 (19)	−0.27281 (12)	0.0452 (3)	
H6	0.4167	0.3387	−0.2924	0.054*	
C7	0.2930 (3)	0.50593 (18)	−0.17026 (11)	0.0392 (3)	
H7	0.4281	0.4977	−0.1211	0.047*	
C8	0.3531 (2)	0.87653 (17)	0.13439 (10)	0.0337 (3)	
C9	0.1943 (3)	0.86476 (19)	0.20396 (10)	0.0396 (3)	
H9	0.0509	0.7884	0.1818	0.048*	
C10	0.2490 (3)	0.96646 (19)	0.30619 (11)	0.0415 (3)	
H10	0.1426	0.9588	0.353	0.05*	
C11	0.4617 (3)	1.07920 (18)	0.33843 (10)	0.0396 (3)	
C12	0.6211 (3)	1.09201 (19)	0.27047 (11)	0.0419 (3)	
H12	0.7643	1.1685	0.293	0.05*	
C13	0.56679 (8)	0.99040 (5)	0.16848 (3)	0.0391 (3)	
H13	0.6744	0.9983	0.1222	0.047*	
Cl1	0.53006 (8)	1.20876 (5)	0.46690 (3)	0.06227 (17)	

### Atomic displacement parameters (Å^2^)

**Table:**

	*U*^11^	*U*^22^	*U*^33^	*U*^12^	*U*^13^	*U*^23^
N1	0.0320 (6)	0.0455 (7)	0.0326 (6)	0.0003 (5)	0.0068 (5)	0.0037 (5)
O1	0.0336 (5)	0.0849 (8)	0.0394 (6)	0.0060 (5)	0.0073 (4)	−0.0005 (5)
C1	0.0337 (7)	0.0404 (7)	0.0347 (7)	0.0024 (5)	0.0045 (5)	0.0076 (6)
C2	0.0328 (7)	0.0340 (7)	0.0345 (7)	−0.0022 (5)	0.0060 (5)	0.0069 (5)
C3	0.0328 (7)	0.0422 (7)	0.0400 (7)	0.0032 (5)	0.0043 (5)	0.0066 (6)
C4	0.0402 (8)	0.0505 (8)	0.0379 (7)	−0.0029 (6)	−0.0035 (6)	0.0075 (6)
C5	0.0487 (8)	0.0474 (8)	0.0342 (7)	−0.0054 (6)	0.0074 (6)	−0.0009 (6)
C6	0.0394 (8)	0.0436 (8)	0.0471 (8)	0.0043 (6)	0.0117 (6)	0.0020 (6)
C7	0.0335 (7)	0.0412 (7)	0.0394 (7)	0.0023 (5)	0.0026 (5)	0.0077 (6)
C8	0.0325 (6)	0.0343 (7)	0.0323 (6)	0.0043 (5)	0.0039 (5)	0.0067 (5)
C9	0.0343 (7)	0.0451 (8)	0.0369 (7)	−0.0042 (6)	0.0026 (5)	0.0097 (6)
C10	0.0404 (7)	0.0510 (8)	0.0333 (7)	0.0031 (6)	0.0084 (6)	0.0113 (6)
C11	0.0442 (8)	0.0378 (7)	0.0317 (7)	0.0065 (6)	0.0004 (6)	0.0043 (5)
C12	0.0369 (7)	0.0385 (7)	0.0441 (8)	−0.0035 (6)	0.0005 (6)	0.0051 (6)
C13	0.0347 (7)	0.0416 (7)	0.0401 (7)	0.0007 (5)	0.0091 (6)	0.0089 (6)
Cl1	0.0764 (3)	0.0615 (3)	0.0352 (2)	−0.0009 (2)	−0.00138 (18)	−0.00280 (18)

### Geometric parameters (Å, °)

**Table:**

N1—C1	1.3560 (18)	C6—H6	0.93
N1—C8	1.4125 (17)	C7—H7	0.93
N1—H1N	0.845 (16)	C8—C13	1.3862 (13)
O1—C1	1.2196 (16)	C8—C9	1.3862 (18)
C1—C2	1.4909 (18)	C9—C10	1.3817 (19)
C2—C3	1.3876 (19)	C9—H9	0.93
C2—C7	1.3885 (19)	C10—C11	1.377 (2)
C3—C4	1.377 (2)	C10—H10	0.93
C3—H3	0.93	C11—C12	1.373 (2)
C4—C5	1.376 (2)	C11—Cl1	1.7402 (14)
C4—H4	0.93	C12—C13	1.3786 (15)
C5—C6	1.379 (2)	C12—H12	0.93
C5—H5	0.93	C13—H13	0.93
C6—C7	1.379 (2)		
			
C1—N1—C8	126.64 (11)	C6—C7—C2	120.02 (13)
C1—N1—H1N	117.7 (11)	C6—C7—H7	120
C8—N1—H1N	115.0 (12)	C2—C7—H7	120
O1—C1—N1	123.02 (12)	C13—C8—C9	119.42 (11)
O1—C1—C2	121.31 (12)	C13—C8—N1	117.74 (10)
N1—C1—C2	115.66 (11)	C9—C8—N1	122.80 (12)
C3—C2—C7	119.04 (12)	C10—C9—C8	120.05 (12)
C3—C2—C1	117.62 (11)	C10—C9—H9	120
C7—C2—C1	123.33 (12)	C8—C9—H9	120
C4—C3—C2	120.51 (12)	C11—C10—C9	119.65 (12)
C4—C3—H3	119.7	C11—C10—H10	120.2
C2—C3—H3	119.7	C9—C10—H10	120.2
C5—C4—C3	120.17 (13)	C12—C11—C10	120.97 (13)
C5—C4—H4	119.9	C12—C11—Cl1	119.21 (11)
C3—C4—H4	119.9	C10—C11—Cl1	119.82 (11)
C4—C5—C6	119.71 (13)	C11—C12—C13	119.40 (12)
C4—C5—H5	120.1	C11—C12—H12	120.3
C6—C5—H5	120.1	C13—C12—H12	120.3
C7—C6—C5	120.53 (13)	C12—C13—C8	120.52 (9)
C7—C6—H6	119.7	C12—C13—H13	119.7
C5—C6—H6	119.7	C8—C13—H13	119.7
			
C8—N1—C1—O1	−1.4 (2)	C1—C2—C7—C6	178.39 (12)
C8—N1—C1—C2	177.27 (12)	C1—N1—C8—C13	−149.06 (12)
O1—C1—C2—C3	28.33 (19)	C1—N1—C8—C9	33.4 (2)
N1—C1—C2—C3	−150.35 (12)	C13—C8—C9—C10	0.24 (18)
O1—C1—C2—C7	−150.27 (14)	N1—C8—C9—C10	177.75 (12)
N1—C1—C2—C7	31.06 (18)	C8—C9—C10—C11	0.0 (2)
C7—C2—C3—C4	−0.8 (2)	C9—C10—C11—C12	−0.1 (2)
C1—C2—C3—C4	−179.47 (12)	C9—C10—C11—Cl1	179.53 (10)
C2—C3—C4—C5	1.0 (2)	C10—C11—C12—C13	0.0 (2)
C3—C4—C5—C6	−0.3 (2)	Cl1—C11—C12—C13	−179.61 (9)
C4—C5—C6—C7	−0.7 (2)	C11—C12—C13—C8	0.18 (17)
C5—C6—C7—C2	1.0 (2)	C9—C8—C13—C12	−0.32 (15)
C3—C2—C7—C6	−0.2 (2)	N1—C8—C13—C12	−177.95 (10)

### Hydrogen-bond geometry (Å, °)

**Table:**

*D*—H···*A*	*D*—H	H···*A*	*D*···*A*	*D*—H···*A*
C9—H9···O1	0.93	2.43	2.9090 (17)	112
N1—H1N···O1^i^	0.845 (16)	2.390 (16)	3.1710 (15)	154.0 (15)
C13—H13···O1^i^	0.93	2.58	3.2507 (11)	129

Symmetry codes: (i) *x*+1, *y*, *z*.
